# Report from the 27th Annual Western Canadian Gastrointestinal Cancer Consensus Conference on Colorectal Cancer, Calgary, Alberta, 26–27 September 2025: Advances in Colon and Rectal Cancer †

**DOI:** 10.3390/curroncol33070437

**Published:** 2026-07-21

**Authors:** Richard Lee-Ying, Sharlene Gill, Adrian Box, Hannah Latour, Scott Strum, Vallerie Gordon, Ralph Wong, Petra Grendarova, Tamara Gimon, Shahid Ahmed, Georgia Geller, Christina Kim, Duc Le, Karen Mulder, James Paul, Branawan Gowrishankar

**Affiliations:** 1 Arthur J.E. Child Comprehensive Cancer Centre, Calgary, AB T2N 4N1, Canada; scott.strum@cancercarealberta.ca; 2 BC Cancer—Vancouver, Vancouver, BC V5Z 4E6, Canada; sgill@bccancer.bc.ca; 3 Alberta Precision Laboratories, Calgary, AB T2N 4N1, Canada; adrian.box@albertaprecisionlabs.ca; 4 Department of Oncology, Faculty of Medicine and Dentistry, University of Alberta, Edmonton, AB T6G 1Z2, Canada; hannah.latour@albertahealthservices.ca; 5 Saskatchewan Cancer Agency, Saskatoon, SK S7N 4H4, Canada; vallerie.gordon@saskcancer.ca (V.G.); shahid.ahmed@saskcancer.ca (S.A.); duc.le@saskcancer.ca (D.L.); 6 CancerCare Manitoba, Winnipeg, MB R2H 2A6, Canada; rwong2@cancercare.mb.ca (R.W.); ckim3@cancercare.mb.ca (C.K.); jpaul@cancercare.mb.ca (J.P.); 7 BC Cancer—Victoria, Victoria, BC V8R 4Z3, Canada; petra.grendarova@bccancer.bc.ca (P.G.); ggeller@bccancer.bc.ca (G.G.); 8 Alberta Health Services, Calgary, AB T2N 2T9, Canada; tamara.gimon@albertahealthservices.ca; 9 Cross Cancer Institute, Edmonton, AB T6G 1Z2, Canada; karen.mulder@cancercarealberta.ca (K.M.); branawan.gowrishankar@cancercarealberta.ca (B.G.)

**Keywords:** colorectal cancer, colon cancer, rectal cancer, metastatic colorectal cancer, adjuvant therapy, immunotherapy, immune checkpoint inhibitors, PI3K pathway mutations, BRAF V600E, deficient mismatch repair, dMMR, microsatellite instability–high, MSI-H, structured exercise intervention, nonoperative management

## Abstract

The 27th annual Western Canadian Gastrointestinal Cancer Consensus Conference (WCGCCC) was held in Calgary, Alberta, on 26–27 September 2025. The WCGCCC is an interactive multidisciplinary conference that was attended by healthcare professionals from across Western Canada (British Columbia, Alberta, Saskatchewan, and Manitoba) who are involved in the care of patients with colorectal cancer. Specialists from the fields of medical and radiation oncology, pathology, surgery, and a family physician in oncology participated in presentations and discussions for the purpose of developing the recommendations presented here. This consensus statement addresses recent advances in the management of colorectal cancer in a Western Canadian context, with respect to adjuvant exercise, as per the CHALLENGE trial, adjuvant Aspirin and PI3K testing, as per the ALASCCA trial, adjuvant immunotherapy, as per the ATOMIC trial, the use of encorafenib and an EGFR inhibitor with chemotherapy, as per the BREAKWATER trial, the use of combination immunotherapy as per the CHECKMATE 8HW trial and optimal strategies for omitting radiation and non-operative management of non-metastatic rectal cancer.

## Simple Summary

The 27th annual Western Canadian Gastrointestinal Cancer Consensus Conference (WCGCCC) was an interactive multidisciplinary conference, that leveraged the insights and experience of healthcare professionals from across Western Canada with expertise in the management of colon and rectal cancer. Consensus statements were made regarding the implications of the CHALLENGE, ALASCCA, ATOMIC, BREAKWATER and CHECKMATE 8HW clinical trials, as well as selective omission of radiation and non-operative management of non-metastatic rectal cancer. The statements reflect the interpretation of recent evidence in a Western Canadian context.

## 1. Terms of Reference

### 1.1. Purpose

The aim of the Western Canadian Gastrointestinal Cancer Consensus Conference (WCGCCC) is to establish evidence-based clinical consensus by engaging specialists from across Western Canada, who are working in the fields of medical and radiation oncology, pathology, surgery, internal medicine and family physicians in oncology. By defining best clinical practice, the participants of the 27th Annual WCGCCC strive to improve the care and clinical outcomes for patients with colorectal cancer.

### 1.2. Participants

The WCGCCC welcomes health professionals from Western Canada who are involved in the care of patients with colorectal cancer (Table 1). Participants are provided the clinical questions in advance that will be addressed during the consensus conference (Table 2). A summary of the consensus statement is available in Table 3.

### 1.3. Target Audience

The recommendations presented here are written for healthcare professionals involved in the care of patients with colorectal cancer.

### 1.4. Basis of Recommendations

The recommendations are based on presentation and discussion of the best available evidence. References are cited where applicable.

**Table 3:** Key takeaway points from the 27th annual WCGCCC.

**Which patients with resected colon cancer should be recommended structured exercise programs (SEPs) to improve outcomes? What key components of a SEP should be offered to patients with resected colorectal cancer?** **A structured exercise program (SEP) should be recommended for patients with resected stage III or high-risk stage II colon cancer who are within 2 to 6 months of completing adjuvant 5-fluorouracil (5FU)-based chemotherapy.** • Patients who are able to participate in a SEP; • Patients who are not already meeting recommended guidelines of >150 min moderate-vigorous or >75 min vigorous physical activity per week; • Extrapolation to other adjuvant colorectal settings (such as stage III rectal cancer) may be justifiable, if resources are available; • The SEP consists of physical-activity-consultant-supervised sessions every 2 weeks for 12 months (behavioral support and exercise supervision); • Supervised sessions may then be monthly in years 2 and 3; • May be in-person or virtual; • Provision of health education materials is also recommended.
**What is the optimal testing strategy for screening for PI3K alterations? What alternative testing strategies can be considered?** **Which patients with resected colorectal cancer should be offered adjuvant ASA? In the absence of PI3K testing, can adjuvant ASA be offered to all cases of resected colorectal cancer?** • Given the significant clinical benefit, patients with stage I-III rectal and stage II-III colon cancer should be offered testing for PI3K pathway alterations; the optimal testing strategy should be informed by local resources and lab capacity, in consultation with pathology. • Next-generation sequencing (NGS) is preferred to identify patients who would benefit from adjuvant low-dose ASA; however, this incurs extensive infrastructure and testing costs. In consultation with molecular pathology, alternative strategies using targeted panel testing may be considered. Currently available PIK3CA (phosphatidylinositol-4,5-bisphosphate 3-kinase catalytic subunit alpha) gene-targeted panels, however, may not encompass the required relevant mutations. • Three years of adjuvant ASA at a dose of 160 mg daily is recommended in patients with stage I-III rectal cancer and stage II-III colon cancer who have PI3K pathway mutations, and who have no contraindications to ASA. • In the absence of testing PI3K alterations, there is no demonstrated benefit of adjuvant ASA in all-comers.
**Is there a role for immune checkpoint inhibitors in addition to adjuvant chemotherapy for patients with resected stage III deficient mismatch repair (dMMR) colon cancer?** **Is there an optimal time for immune checkpoint inhibition pre-, post- or perioperatively? Is there a role for chemotherapy de-escalation?** • The addition of atezolizumab to adjuvant oxaliplatin-based doublet chemotherapy is recommended in patients with resected stage III (node-positive) dMMR colon cancers. • Neoadjuvant/perioperative therapy has shown promising efficacy; however, phase III trials are required to determine optimal sequencing.
**What is the optimal first-line therapy for patients with BRAF V600E-mutant metastatic colorectal cancer?** • BRAF-directed inhibition with encorafenib, in combination with an epidermal growth factor receptor (EGFR) inhibitor and folinic acid, fluorouracil, and oxaliplatin (FOLFOX) is the preferred first-line therapy for patients with BRAF V600E and proficient mismatch repair (pMMR) colorectal cancer. • Patients ineligible for oxaliplatin-based chemotherapy can be considered for first-line encorafenib and an EGFR inhibitor. • Timely BRAF ascertainment is needed to direct first-line management and should be available at time of medical oncology consultation. • If BRAF status is unknown, it is reasonable to bridge with a doublet and switch to FOLFOX + encorafenib when BRAF V600E status is confirmed. • Immunotherapy is the preferred first-line treatment for patients with dMMR and BRAF V600E colorectal cancer.
**What is the preferred immunotherapy approach for patients with dMMR metastatic colorectal cancer: single-agent or combination immunotherapy?** • Combination immunotherapy with nivolumab and ipilimumab is the preferred option. • Single-agent (programmed cell death protein-1 (PD-1)) immune checkpoint inhibition may be an option in selected patients.
**In which patients with localized rectal cancer can radiotherapy be safely omitted without compromising outcomes?** • Multidisciplinary discussion is recommended to determine optimal management for patients with locally advanced rectal cancer when there are overlapping criteria for different management strategies. • Patients who can proceed straight to surgery (i.e., omission of neoadjuvant treatment), including: ○ Patients with pMMR cT1N0; cT2N0; or low-risk cT3N0 adenocarcinoma who intend to proceed with operative management. • Patients who can be treated with neoadjuvant chemotherapy alone and who meet all of the following criteria: ○ pMMR cT2—cT3a/b N0–1 (<4 LN > 1 cm), mid- to upper rectal adenocarcinoma (>5 cm from anal verge or sphincter-sparing) magnetic resonance-assessed circumferential resection margin (mrCRM) ≥ 3 mm; ○ Patients who intend to proceed with operative management; ○ Patients treated with neoadjuvant chemotherapy who achieve > 20% decrease in the size of the primary tumor on imaging and endoscopic evaluation. • Patients in a postoperative setting who did not receive neoadjuvant radiotherapy (RT), with good quality total mesorectal excision (TME) and without high-risk features such as circumferential resection margin (CRM) positivity, pT4b, pN2, extracapsular spread, and proximity to the mesorectal fascia (MRF). • Patients with dMMR rectal cancer who can be treated with immunotherapy and achieve complete response.
**What are the key criteria to guide safe implementation of a non-operative watch-and-wait strategy in rectal cancer?** • Patient counselling is crucial as well as a multidisciplinary review of rectal cancer cases to determine the optimal neoadjuvant approach and whether non-operative management (NOM) would be feasible. The involvement of an experienced surgeon is recommended when considering NOM. • The best evidence for NOM is in those who have had a TNT approach, preferably with long-course chemoradiation followed by consolidation chemotherapy. • Patients should be counseled with a focus on potential risks and benefits and the likelihood of NOM, as well as alternative strategies. • MRI, computerized tomography chest, abdomen and pelvis (CT-CAP), digital rectal exam (DRE) and flexible sigmoidoscopy should be completed 8 weeks (±4 weeks) post-completion of TNT if considering NOM. • Criteria for NOM include cCR as assessed by a colorectal surgeon with experience in NOM (near-complete requires follow-up). • Those on NOM should be followed with intensive surveillance including regular clinical exam, endoscopy, MRI, and CT-CAP according to an established protocol, with emphasis in the initial 2 years and continuing for at least 5 years.

## 2. Question(s) 1—Which Patients with Resected Colon Cancer Should Be Recommended Structured Exercise Programs (SEPs) to Improve Outcomes? What Key Components of a Structured Exercise Program Should Be Offered to Patients with Resected Colorectal Cancer?

### Summary of Evidence

**Exercise as an Adjuvant Strategy in Early-stage Colon Cancer: The CHALLENGE Trial**

Regular physical activity is increasingly recognized as an important contributor to improve outcomes for individuals treated for colon cancer. A growing body of epidemiologic evidence shows that higher levels of post-treatment physical activity are associated with reduced recurrence rates and improved survival [1,2]. These benefits are believed to arise from several interconnected biological mechanisms: enhanced insulin sensitivity, reductions in systemic inflammation, improved immune system engagement, and gains in overall cardiopulmonary fitness [1]. These findings create a strong rationale for evaluating whether structured physical activity could be evaluated in a randomized study to improve survival outcomes.

The Canadian Cancer Trials Group (CCTG) CO.21 (CHALLENGE) trial is a multinational, randomized phase III clinical study evaluating whether a structured exercise program can improve outcomes for people with resected stage III or high-risk stage II colon cancer who have completed adjuvant chemotherapy, to address the question of whether regular supervised physical activity reduces the risk of cancer recurrence and improves long-term survival [3]. Participants included adults with resected stage III or high-risk stage II colon cancer who had completed adjuvant fluoropyrimidine-based chemotherapy within the previous two to six months. To ensure that the intervention targeted individuals that were likely to benefit, eligibility required that participants were not already meeting physical activity guidelines (defined as greater than 150 min of moderate-to-vigorous activity or 75 min of vigorous activity per week). Baseline fitness was formally assessed through standardized tests such as a 6 min walk or multi-stage exercise test.

The intervention consisted of a three-year, structured exercise program grounded in both physical training and behavioral support:

- Every 2 weeks for 12 months: Supervised sessions with a physical activity consultant, incorporating exercise guidance and behavioral coaching.
- Years 2 and 3: Monthly follow-up sessions, offered virtually or in person.
- Exercise prescription: Aerobic and resistance training tailored to fitness level and delivered progressively over time.

The program used the Theory of Planned Behavior framework to enhance motivation, confidence and long-term adherence [4]. Participants received education materials and coaching on goal setting, problem solving, and building self-efficacy.

Participants were randomized 1:1 to either the group with structured exercise program (SEP) and health education materials (HEM), or HEM alone. Both groups received standard oncologic follow-up, with the intervention arm receiving additional supervised exercise and behavioral counselling. The primary endpoint was disease-free survival (DFS), with secondary endpoints of overall survival (OS), physical fitness, adherence, quality of life and behavioral change. At a median follow-up of 7.9 years, DFS was significantly longer in the SEP group than in the HEM group. The 5-year DFS was 80% in the SEP group and 74% in the HEM group (HR 0.72, 95% confidence interval (CI) 0.55–0.94; *p* = 0.017). The 8-year OS was 90% in the SEP and 83% in the HEM group (HR 0.63, 95% CI 0.43–0.94; *p* = 0.022). Musculoskeletal adverse events occurred more often in the SEP than in the HEM group (in 19% vs. 12% of patients).

The CHALLENGE trial provides compelling level 1 evidence that a structured, behaviorally supported exercise program provides a clinically meaningful improvement in DFS and OS after curative-intent treatment for colon cancer. The core components of the recommended program include regular supervised sessions, extended behavioral follow-up over several years, and access to health education resources designed to support long-term adherence. Despite the relatively low cost, implementing exercise programs within cancer systems is challenging because they do not align with traditional funding structures. To address these barriers, several strategies are proposed. Cancer centers may employ dedicated exercise professionals or integrate services with community partners such as wellness organizations, rehabilitation programs, prehabilitation groups, and local gyms. There is also potential to collaborate with existing chronic disease programs like cardiac rehabilitation or diabetes education, leveraging shared infrastructure and multidisciplinary expertise.

## 3. Question(s) 2a—What Is the Optimal Testing Strategy for Screening for PI3K Alterations? What Alternative Testing Strategies Can Be Considered?

**Question(s) 2b—Which patients with resected colorectal cancer should be offered adjuvant ASA? In the absence of PI3K testing, can adjuvant ASA be offered to all cases of resected colorectal cancer?**

### 3.1. Summary of Evidence—Question 2a

**Precision Adjuvant Therapy for Phosphatidylinositol 3-Kinase (PI3K) Pathway Mutated Colorectal Cancer: The ALASCCA Trial**

Based on the results of the Low-Dose Aspirin for PI3K-Altered Localized Colorectal Cancer (ALASCCA) trial, it was recommended that early-stage colorectal carcinoma (CRC) be tested for actionable mutations in the *PIK3CA* pathway [5]. The trial findings have important implications for molecular testing strategies and implementation in routine clinical practice. These implications and practical considerations were reviewed and discussed by the WCGCCC group.

In order to best achieve alignment with ALASCCA, molecular testing for the following genes should be considered: *PIK3CA*, *PTEN*, *AKT1*, and *PIK3R1*. Based on data from the ALASCCA trial, the most comprehensive assessment of the above genes will require large panel NGS-based testing (full gene sequencing) of early-stage (I–III) CRC cases. This testing would be in addition to standard molecular testing required for advanced stage CRC (*BRAF*, *MSI*, *MMR*) and would represent a significant increase in testing costs to most health regions. It is commonly discussed that alternative smaller, readily available commercial panels containing the most common PIK3CA mutations (e.g., E542, E545, H1047) in colorectal cancer could be used as a cost savings measure but would miss an estimated 12–20% of possible CRC cases. Additional considerations for testing included that testing per ALASCCA would necessitate testing of colon biopsies, which represents limited tissue and increases the risk of failed testing, and that most standard commercial large-scale NGS panels would likely not include full sequencing of all the above genes (e.g., *PIK3R1*).

These recommendations should be interpreted within the context of local oncology practice patterns, regional molecular testing infrastructure, and available laboratory funding and personnel resources.

### 3.2. Summary of Evidence—Question 2b

Multiple studies have shown a beneficial effect of aspirin in the primary prevention of colorectal cancer and adenomas [6,7,8,9,10,11]. Several retrospective studies have also demonstrated that aspirin may reduce the risk of colorectal cancer recurrence [12,13]. It has been proposed that aspirin may block steps in the PI3K pathway, leading to apoptosis of cancer cells [14,15].

A retrospective study published by Liao and colleagues examined data from two prospective cohort studies that included patients with stage I-IV colorectal cancer [16]. *PIK3CA* mutation data and information on aspirin use were available for 964 patients [16]. In patients with *PIK3CA*-mutated colorectal cancers, regular use of aspirin after colorectal cancer diagnosis was associated with a statistically significant improvement in colorectal cancer-specific survival (HR 0.18, 95% CI 0.06–0.61; *p* < 0.001) and OS (HR 0.54, 95% CI 0.31–0.94; *p* = 0.01) [16]. The same benefit was not observed for patients lacking a *PIK3CA* mutation [16].

A randomized phase III clinical trial by Chia and colleagues randomized patients with Dukes’ C or high-risk Dukes’ B colon cancer, or Dukes’ B or C rectal cancer, to receive 200 mg of aspirin or placebo, once daily for three years after completing adjuvant chemotherapy [17]. This trial failed to show a statistically significant difference in DFS (primary endpoint) or OS (secondary endpoint) [17]. Estimated DFS at 5 years was 77.0% (95% CI 73.6–80.0%) for the aspirin group and 74.8% (95% CI, 71.3–77.9%) for the placebo group (HR 0.91, 95% CI 0.73–1.13; *p* = 0.38) [17]. While the HR for OS seemed to favor aspirin, this result did not meet statistical significance (HR 0.75, 95% CI 0.53–1.07, *p* = 0.11) [17].

A later trial by Güller and colleagues randomized patients with stage II or III resected colorectal cancer with an activating *PIK3CA* mutation, to receive aspirin 100 mg once daily or placebo for 3 years in the adjuvant setting [18]. This trial was closed early due to financial constraints, allowing for the randomization of 112 patients [18]. The trial failed to show a statistically significant difference in DFS at three years (88.3% (aspirin arm), 82.4% (placebo arm) HR 0.57, 90% CI 0.27–1.22; *p* = 0.11) or time to recurrence (HR 0.49, 90% CI 0.21–1.19; *p* = 0.089) [18].

The recently published ALASCCA trial was a phase III clinical trial that randomized patients with stage I–III rectal cancer or stage II–III colorectal cancer to receive adjuvant aspirin 160 mg daily or placebo for three years [5]. Patients were required to have a somatic alteration in the PI3K pathway (exon 9 or 20 mutations (Group A) or *PIK3R1*, *PTEN* or other *PIK3CA* mutations (Group B)) to be included [5]. The study demonstrated a significant reduction in colorectal cancer recurrence rate at three years in patients with Group A mutations randomized to aspirin compared to placebo (7.7% vs. 14.1%) (HR 0.49, 95% CI 0.24–0.98; *p* = 0.04) [5]. The same trend was observed in patients with Group B mutations (7.7% vs. 16.8%) (HR 0.42, 95% CI 0.21–0.83) [5]. The secondary endpoint of DFS was also improved for patients randomized to aspirin in both groups (88.5% vs. 81.4% (Group A), (HR 0.61, 95% CI 0.34–1.08) and 89.1 vs. 78.7% (Group B), (HR 0.51, 95% CI 0.29–0.88)) [5]. The most common adverse events were hematologic or gastrointestinal disorders. Four patients had a serious adverse event that was felt to be aspirin-related, and these included an allergic reaction, gastrointestinal bleed, subarachnoid hemorrhage and spontaneous hematoma [5]. Four patients experienced death, with one event considered to be possibly related to aspirin use [5].

## 4. Question(s) 3—Is There a Role for Immune Checkpoint Inhibitors in Addition to Adjuvant Chemotherapy for Patients with Resected Stage III Deficient Mismatch Repair (dMMR) Colon Cancer? Is There an Optimal Time for Immune Checkpoint Inhibition Pre-, Post- or Perioperatively? Is There a Role for Chemotherapy De-Escalation?

### Summary of Evidence

**Immune Checkpoint Inhibition in dMMR Stage III Colon Cancer: the ATOMIC Trial and Beyond**

The mismatch repair (MMR) mechanism is a post-replicative DNA repair pathway tasked with correcting mismatched bases or small insertions and deletions [19]. It is classically associated with the pan-solid tumor microsatellite instability–high (MSI-H) hypermutable phenotype. In the CRC setting specifically, dMMR/MSI-H tumors are disproportionally right-sided, frequently poorly differentiated, and enriched for *BRAF* V600E alterations [20,21]. Prevalence of this phenotype varies by stage but is generally observed in roughly 20% of patients with stage II CRC disease, 12% of stage III disease, and approximately 5% of stage IV disease [22]. Historically, the role of adjuvant cytotoxic chemotherapy in MSI-H localized disease has been context-dependent. Several studies have demonstrated limited utility of single-agent fluoropyrimidine adjuvant therapy in stage II MSI-H disease, where fluoropyrimidines may offer little benefit, and in some series may actually be harmful [23,24]. By contrast, in the stage III setting, pooled analyses have shown the efficacy of doublet-based chemotherapy in improving outcomes in adjuvant regimens for stage III disease and constitute a standard of care in this space [25,26,27,28].

In the era of immune checkpoint inhibitor (ICI) therapy, practice-changing applications have been found in patients with metastatic dMMR/MSI-H CRC. Studies such as the pivotal phase II study showing high objective response rates in dMMR colorectal tumors relative to their MMR-proficient/MSI-stable CRC counterparts helped provide evidence for their efficacy in this space [29]. ICIs are now considered the standard of care in the first-line setting for advanced dMMR/MSI-H CRC, born in part from data of the KEYNOTE-177 and CHECKMATE-8HW clinical trials [30,31]. Clinical observations of increased efficacy, and comparatively lower rates of grade 3–5 adverse events relative to chemotherapy, have driven the enthusiasm to study the applicability of ICIs in the curative-intent MSI-H colon cancer setting for patients with localized disease. This included whether or not ICIs should be combined with adjuvant chemotherapy for resected stage III dMMR colon cancer to improve clinical outcomes, and whether or not there is an optimal timing for ICI delivery in the curative-intent colon cancer space (pre-, post- or perioperative). The sections below will help to provide a consensus perspective on these crucial questions.

The recent randomized phase III ATOMIC trial (NCT02912559) directly tested whether adding PD-L1 blockade to standard oxaliplatin-based adjuvant chemotherapy improves outcomes in stage III dMMR colon cancer [32]. ATOMIC randomized patients after complete R0 resection to receive either mFOLFOX6 (modified FOLFOX-6) alone for 6 months, or mFOLFOX6 combined with atezolizumab (given concurrently for six months and thereafter continued as monotherapy for an additional six months), with DFS as the primary endpoint [32]. At a median follow-up of 40.9 months, the 3-year disease-free survival was 86.3% (95% CI 81.8–89.8) in the atezolizumab-mFOLFOX6 group, as compared with 76.2% (95% CI 70.9–80.6) in the mFOLFOX6 group (HR for disease recurrence or death, 0.50; 95% CI 0.35–0.73; *p* < 0.001) [32]. The trial met its primary endpoint, and the result was statistically significant. The benefit of adjuvant atezolizumab was consistent across prespecified subgroups, including low- versus high-risk disease, proximal versus distal primaries, and older versus younger patients [32]. Grade ≥3 treatment-related adverse events were more frequent in the atezolizumab arm (84.1% vs. 79.1%), but no unexpected safety signals were reported [32]. ATOMIC provides a high level of randomized evidence to support the addition of PD-L1 blockade into oxaliplatin-based adjuvant chemotherapy, significantly improving DFS in stage III dMMR colon cancer, and reducing the risk of recurrence by roughly half in the reported analysis [32]. The magnitude of the benefit supports the adoption of chemo-immunotherapy (mFOLFOX6 + atezolizumab followed by atezolizumab monotherapy per study schema) as a new adjuvant standard for this population [32]. These data extend the translational logic established in the metastatic setting that MSI-H biology is predictive of high sensitivity to PD-1/PD-L1 axis blockade.

Important caveats from this trial remain. First, although the ATOMIC results are now supported by a full peer-reviewed publication that provides detailed efficacy and safety data, longer follow-up remains important to better define the durability of benefit and mature survival outcomes such as OS [32]. Second, several important clinical questions remain. For example, whether chemotherapy can be safely de-escalated, such as from 6 months of doublet chemotherapy to 3 months in lower-risk patients, is unknown. Whether or not a full year of adjuvant ICI is required is also unclear. Even though most adverse events were considered manageable, the balance of DFS benefit with the added toxicity in the interventional arm must be individualized, particularly for frail patients or those with comorbid autoimmune disease. Furthermore, it is uncertain whether or not these results can successfully be extrapolated to stage dMMR/MSI-H rectal cancer or stage II colon cancer. Lastly, additional adjuvant interventions such as ASA in patients with PI3K alterations or structured exercise that have found efficacy in improving outcomes in the adjuvant colon cancer space will need to be assessed if they also apply to the dMMR population that receives adjuvant ICI [3,5]. Extending adjuvant atezolizumab to patients outside of those who were studied in the ATOMIC trial should only be done under the purview of a clinical trial. Finally, modern tools such as biomarkers (e.g., use of circulating tumor DNA to identify patients at very low risk who might derive limited benefit from adjuvant intensification), may offer a promising area of research to further personalize adjuvant treatment approaches in this unique population.

Taken altogether, ATOMIC supports a new standard of care in this population through the addition of adjuvant ICI to oxaliplatin-based chemotherapy. Adjuvant ICI improves DFS in resected stage III dMMR colon cancer and should be considered in guideline recommendations, with the understanding that mature survival analyses will further inform practice.

Building upon the successes found in the adjuvant setting, biologic rationale and early clinical data has spurred interest in the neoadjuvant or perioperative immunotherapy in the treatment of dMMR/MSI-H colon cancer. Mechanistically, delivering ICI prior to tumor resection allows exposure of the immune system to a larger repertoire of neoantigens, and maintains intact tumor-draining lymph nodes. This allows enhanced dendritic cell-mediated priming, expansion of tumor-specific T-cell clones, epitope spreading, and the generation of more robust systemic anti-tumor immunity that can more effectively target micro-metastatic disease [33,34]. Clinical trial data corroborates the efficacy of these concepts in CRC. The NICHE-1 and NICHE-2 trials demonstrated high pathologic response rates after even short courses of neoadjuvant nivolumab plus ipilimumab in localized dMMR colon cancer [35,36]. In NICHE-2, major pathologic response rates exceeded 90%, and pathologic complete response (pCR) rates were observed in approximately two-thirds of patients, with very few surgical delays and no early recurrences reported in early follow-up [36]. Single-agent neoadjuvant PD-1 therapy has also yielded high pCR rates in smaller series, and randomized and single-arm trials [37,38] have reported rapid and deep responses after one to a few cycles of pembrolizumab, with pCR rates that are clinically meaningful even in stage III disease. In rectal dMMR disease, single-agent PD-1 blockade produced striking organ-sparing complete clinical responses (cCR) in a small prospective series, underscoring the potential of neoadjuvant immunotherapy in the lower gastrointestinal tract [39].

These data raise the question of whether neoadjuvant or perioperative ICI can supplant or de-escalate adjuvant chemotherapy. Advantages of a preoperative approach include superior immune priming, earlier eradication of occult systemic disease, and tissue-based assessment of response that may guide subsequent therapy. Disadvantages and unknowns include potential risks of immunotherapy-related perioperative morbidity, the need for careful coordination of multidisciplinary care to avoid surgical delay, and limited long-term survival data from randomized trials comparing neoadjuvant strategies with standard adjuvant approaches. Importantly, while studies such as NICHE-2 show promising pathologic responses, randomized evidence demonstrating an improvement in DFS or OS with neoadjuvant immunotherapy versus adjuvant chemo-immunotherapy is currently lacking.

Therefore, although neoadjuvant ICI produces deep and often rapid responses in dMMR tumors and may be immunologically optimal, the current evidence base supports adjuvant chemo-immunotherapy (as per ATOMIC) from phase III clinical trial data and should be considered the current standard for resected stage III disease. Neoadjuvant or perioperative immunotherapy represents a highly promising strategy that should ideally be studied within the context of clinical trials. Case-by-case decisions on using neoadjuvant immunotherapy should be discussed in a multidisciplinary setting. Future randomized perioperative trials and longer follow-up from existing neoadjuvant cohorts will be required to define the definitive sequencing strategy.

The integration of ICIs into curative-intent treatment for dMMR/MSI-H colon cancer is now supported by compelling clinical and biological data. ATOMIC provides pivotal randomized evidence that adding anti-PD-L1 to mFOLFOX6 improves DFS in stage III disease and should alter adjuvant practice pending full study publication. Neoadjuvant studies demonstrate high pathologic responses and suggest that earlier immune priming may yield durable systemic control; however, data from randomized trials comparing clinical outcomes of neoadjuvant/perioperative strategies with adjuvant chemo-immunotherapy are expected. Outstanding questions include the optimal duration of adjuvant ICI, the necessity and duration of oxaliplatin if adjuvant benefit is primarily immune-mediated, the role of ctDNA for treatment selection and de-escalation, and the applicability of these strategies to *polymerase epsilon* (*POLE*)-mutant or other hypermutated non-MSI tumors. As data mature, personalization of timing and intensity of immunotherapy will likely be the next evolutionary step in curing dMMR/MSI-H colon cancer.

## 5. Question(s) 4—What Is the Optimal First-Line Therapy for Patients with BRAF V600E-Mutant Metastatic Colorectal Cancer?

### Summary of Evidence

**BREAKWATER—BRAF-Directed Therapy in BRAF V600E-mutated Metastatic Colorectal Cancer**

*BRAF* V600E mutations in colorectal cancer occur in approximately 8–12% of patients [40]. In patients with metastatic disease treatment with *BRAF* inhibitors has shown a benefit in the second-line setting. Their activity in conjunction with chemotherapy as well as their safety has been reported in previous publications for other indications. Chemotherapy in the first-line setting for *BRAF*-mutated colorectal cancer is of limited benefit and survival is poor.

The BREAKWATER trial is an open-label, multicenter, phase 3 study in first-line *BRAF* V600E mutant metastatic colorectal cancer patients and was designed to compare encorafenib with cetuximab (EC) in arm A, encorafenib, cetuximab and mFOLFOX6 chemotherapy in arm B, and standard of care chemotherapy in arm C [41]. Dual primary endpoints were progression-free survival (PFS) and overall response rate by blinded independent central review (BICR), and the key secondary endpoint was OS, comparing EC with FOLFOX 6 to standard of care [41].

Significantly longer PFS was observed with EC + mFOLFOX6 compared to the standard care (median, 12.8 vs. 7.1 months; HR (progression or death) 0.53, 95% CI 0.41–0.68; *p* < 0.001) [41]. EC alone in arm A was stopped after 158 patients were enrolled and the remaining patients were randomized to arm B or C after a protocol amendment [41]. Median PFS in the EC plus modified FOLFOX 6 arm (arm B) was 12.8 months compared with 7.1 months for the standard of care arm and 6.8 months for the EC alone arm [41]. In an interim analysis, OS was significantly longer with EC + mFOLFOX6 than with standard care (median, 30.3 vs. 15.1 months; HR (death) 0.49, 95% CI 0.38–0.63; *p* < 0.001) [41]. Subgroup analysis found benefit in all subgroups of patients, stratified by age, sex, Eastern Cooperative Oncology Group (ECOG) performance status 0 or 1, number of organs involved, liver metastases or no liver metastases, as well as primary tumor location in the left or right side of the colon [41].

BREAKWATER showed a statistically significant and clinically meaningful benefit in overall response rates by BICR, as well as significantly improved progression-free and overall survival, with EC plus mFOLFOX 6 versus standard of care [41,42]. These findings support EC plus mFOLFOX6 as a new first-line treatment standard for patients with BRAF V600E-mutated metastatic colorectal cancer [41,42].

BREAKWATER cohort 3 randomized patients to EC + FOLFIRI vs. FOLFIRI ± bevacizumab. The objective response rate was 64.4% vs. 39.2%, (odds ratio (OR) 2.76, 95% CI 1.42–5.35; *p* < 0.001), suggesting that FOLFIRI is a reasonable alternative chemotherapy backbone. Survival data, however, is still immature from this study [43].

## 6. Question(s) 5—What Is the Preferred Immunotherapy Approach for Patients with dMMR Metastatic Colorectal Cancer: Single-Agent or Combination Immunotherapy?

### Summary of Evidence

**Optimizing Immunotherapy in dMMR Metastatic Colorectal Cancer: CheckMate-8HW**

dMMR/MSI-H tumors account for approximately 10–15% of all colorectal cancers. Among patients with stage IV disease, however, the prevalence is lower ranging from 4 to 6% [44]. These tumors are characterized by a high mutational burden, resulting in the generation of numerous mutation-derived neoantigens that provoke robust anti-tumor immune responses. This immune activation is reflected by increased infiltration of CD8-positive tumor-infiltrating lymphocytes within the tumor microenvironment. As a result, dMMR/MSI-H colorectal cancers are particularly sensitive to immune checkpoint inhibition. Although dMMR/MSI-H status is associated with a favorable prognosis in early-stage disease, stage IV tumors often demonstrate more aggressive behavior and inferior survival outcomes compared with pMMR/microsatellite stable (MSS) colorectal cancers. Historically, these tumors have shown limited responsiveness to systemic chemotherapy.

KEYNOTE-177 was a phase III trial that randomized treatment-naïve patients with dMMR/MSI-H metastatic colorectal cancer to receive either pembrolizumab at a dose of 200 mg every three weeks or investigator’s choice of chemotherapy (5FU, folinic acid, and irinotecan (FOLFIRI) or FOLFOX, with or without bevacizumab) [45]. At a median follow-up of 73 months, pembrolizumab demonstrated improvement in OS compared with chemotherapy (median OS 77.5 vs. 36.7 months (HR 0.73, 95% CI 0.53–0.99); five-year OS rates were 54.8% vs. 44.2%). PFS was also improved (median 16.5 vs. 8.2 months (HR 0.60, 95% CI 0.45–0.79); five-year PFS 34.0% vs. 7.6%) [30]. Pembrolizumab was better tolerated than chemotherapy, with lower rates of grade 3–5 treatment-related toxicity (21.6% vs. 67.1%) and clinically meaningful improvements in European Organization for Research and Treatment of Cancer Quality of Life Questionnaire Core 30 (EORTC QLQ-C30) global health status/quality of life (GHS/QOL) scores with pembrolizumab versus chemotherapy (between-group least squares mean (LSM) difference 8.96 [95% CI 4.24–13.69]; two-sided nominal *p* = 0.0002). Median time to deterioration was longer with pembrolizumab versus chemotherapy for GHS/QOL (HR 0.61, [95% CI 0.38–0.98]; one-sided nominal *p* = 0.019), physical functioning (0.50 [95% CI, 0.32–0.81]; one-sided nominal *p* = 0.0016), social functioning (0.53 [95% CI 0.32–0.87]; one-sided nominal *p* = 0.005), and fatigue scores (0.48 [95%, CI 0.33–0.69]; one-sided nominal *p* < 0.0001) [46].

CheckMate H8W was an open-label phase III trial that enrolled patients with stage IV dMMR/MSI-H colorectal cancer across multiple lines of therapy. Patients were randomized in a 2:2:1 ratio to receive either four cycles of nivolumab (240 mg) plus ipilimumab (1 mg/kg) every three weeks, followed by maintenance nivolumab 480 mg every four weeks; nivolumab monotherapy; or investigator’s choice of chemotherapy (FOLFIRI or FOLFOX with or without bevacizumab or cetuximab) until disease progression or unacceptable toxicity [31,47]. Nivolumab plus ipilimumab treatment showed significant and clinically meaningful improvement in PFS versus nivolumab (HR 0.62, 95% CI 0.48–0.81; *p* = 0.0003). Median PFS was not reached with nivolumab plus ipilimumab. OS data remain immature and follow-up is ongoing. Grade 3–4 treatment-related adverse events were more frequent with combination immunotherapy compared with nivolumab monotherapy (22% vs. 14%), though no new safety signals were identified [47].

When compared with chemotherapy, nivolumab plus ipilimumab also demonstrated superior PFS among 303 patients with treatment-naïve disease (median not reached vs. 6 months; two-year PFS 63% (95% CI, 56–70) vs. 15% (95% CI, 7–25)) and among 255 patients with treatment-naïve, centrally confirmed dMMR/MSI-H tumors (median not reached vs. 6 months; two-year PFS 72% (95% CI, 64–79) and 14% (95% CI, 6–25)) [31]. These benefits were again observed across all clinically relevant subgroups. OS data are not yet mature, and follow-up is ongoing. Grade 3–4 treatment-related toxicities were lower with combination immunotherapy than with chemotherapy (23% vs. 48%), with no new toxicity profiles identified.

Based on the available evidence from these studies, first-line therapy with nivolumab plus ipilimumab is favored due to its substantial improvement in PFS and acceptable toxicity profile. For patients who decline combination immunotherapy or are not considered suitable candidates, pembrolizumab monotherapy represents an appropriate alternative.

## 7. Question(s) 6—In Which Patients with Localized Rectal Cancer Can Radiotherapy Be Safely Omitted Without Compromising Outcomes?

### Summary of Evidence

**Radiotherapy in Rectal Cancer: Can It Be Safely Omitted in Selected Patients?**

Neoadjuvant RT in patients with locally advanced rectal adenocarcinoma decreases the risk of local recurrence (LR), even in the era of TME, with the HR 0.59, (95% CI 0.48–0.72) for LR compared with surgery alone. RT improves tumor resectability, reduces the risk of positive margins, allows for tumor downstaging, and has the potential for sphincter/rectum preservation [48,49,50,51,52,53,54,55,56].

There are many reasons to avoid RT, however, including the length of treatment, acute and late radiation toxicity, infertility and menopause in younger patients, increased toxicity in people with autoimmune diseases such as inflammatory bowel diseases, lupus and scleroderma, and a risk of secondary malignancies. Attempts have been made to determine which patients with rectal adenocarcinoma can safely forgo radiation without compromising their cancer outcomes and to identify alternative treatment regimens.

Traditionally, indications for neoadjuvant radiation therapy in rectal adenocarcinoma included patients with T3 and T4 tumors and/or lymph-node-positive disease. As there was no evidence-based neoadjuvant alternative to RT, the aim was to identify patients with a sufficiently low risk of local and regional recurrence who could safely proceed straight to surgery and avoid neoadjuvant therapy altogether. The European Society for Medical Oncology (ESMO) 2017 guidelines listed patients with early disease, which included cT3a/bcN0 tumors in the mid- or high-rectum with clear MRF and no extramural venous invasion (EMVI), as suitable for an upfront TME [57]. In addition, patients with intermediate disease (cT3a/b very low tumors with clear levators and MRF; cT3a/b cN1–2 tumors in the mid- to high-rectum with no EMVI and no evidence of extranodal extension) could proceed with upfront TME, if a good quality surgery could be assured [57]. On the other hand, the American Society for Radiation Oncology (ASTRO) 2021 guidelines generally recommended neoadjuvant radiation in patients with stage II and III rectal cancer and suggested consideration of omission of neoadjuvant radiation only in patients with lower risk (cT3a/b cN0 > 10 cm from the anal verge and magnetic resonance imaging (MRI)-assessed mrCRM ≥ 2 mm, with no MRI-assessed EMVI (mrEMVI)) as suitable for upfront surgery [58].

More recently, the PROSPECT trial led to a paradigm change in neoadjuvant therapy in patients with rectal cancer [59]. PROSPECT was a multicenter, unblinded, non-inferiority, randomized controlled trial (RCT) of neoadjuvant FOLFOX chemotherapy in the experimental arm, with chemoradiotherapy given only if the primary tumor decreased in size by <20% after six cycles, or if FOLFOX was discontinued because of side effects. Patients who were eligible for enrollment in PROSPECT included patients with CT2N1, T3N0, or T3N1, with an ECOG performance status of 0–2 and tumors deemed appropriate for sphincter-sparing surgery [59]. Patients with T4 tumors, N2 disease, tumors within 3 mm of the radial margin and tumors requiring an abdominal perineal resection (APR) were excluded. Overall, a total of 1194 patients were randomized and 1128 started treatment between June 2012 and December 2018. Eighty-five percent of participants had mid- to high-rectal tumors. FOLFOX was found to be non-inferior with respect to the primary outcome, which was DFS at 5 years [59]. In addition, secondary outcomes including 5-year overall OS (HR 1.04, 95% CI 0.74–1.44) and local recurrence-free survival (LRFS) (HR 1.18, 95% CI 0.44–3.16) were not statistically different between the FOLFOX and chemoradiation arms [59]. A total of 9.1% of participants received selective chemoradiotherapy (CRT) in the FOLFOX group, because of either insufficient response (6.5%) or failure to complete chemotherapy. Rates of LR at 5 years were <2% in both groups and pCR rates (21.4%, FOLFOX group v 24%, CRT group) were not statistically different (HR 1.18, 95% CI 0.44–3.16). While initial grade 3+ toxicity was higher in the FOLFOX group, there was no significant difference in patient-reported quality of life [59,60].

Other recent clinical trials exploring similar treatment paradigm included CONVERT and FORWAC clinical trials [61,62,63,64]. CONVERT was a phase III RCT of neoadjuvant capecitabine and oxaliplatin (CAPOX) compared to neoadjuvant CRT and enrolled 663 participants with cT2N1 or cT3–4a locally advanced rectal cancer with uninvolved MRF [61]. It showed no significant difference in R0 resection rate, pCR, grade 3 to 4 toxicity, sphincter preservation rate and postoperative complications [61]. Three-year locoregional recurrence free survival was 97.4% in the CRT arm vs. 96.3% in the CAPOX arm (HR 1.08, 95% CI 0.46–2.54) [62]. While non-inferiority of neoadjuvant chemotherapy alone was not confirmed, the incidence of LR in both arms was very low and neoadjuvant chemotherapy alone resulted in comparable DFS and OS [62].

FOWARC was a phase III trial which randomized a total of 495 patients with stage II–III rectal cancers into three arms: fluorouracil plus radiotherapy, mFOLFOX6 plus radiotherapy, or mFOLFOX6 alone, followed by surgery and adjuvant chemotherapy [63,64]. Participants had T3 to T4N0, or T1 to T4N1–2, and 33% of patients had MRF involvement. There was no statistical difference in OS, DFS and LR, even with a 10-year update [64]. There was a statistically higher pCR in the chemoradiation group, compared to chemotherapy alone (OR 0.428, 95% CI 0.237– 0.776; *p* = 0.005) [64].

Results of these trials led to changes in consensus recommendations and updated guidelines within the last 2 years. The American Society of Clinical Oncology (ASCO) published their updated guidelines for the management of locally advanced rectal cancer in 2024 [65]. These guidelines focus on management of clinical T3 or T4 rectal tumors, and/or lymph-node-positive non-metastatic disease [65]. For patients with pMMR mid- to upper-rectal cancers with >5 mm of extramural invasion, and without high-risk factors for LR and/or distant metastases (defined as T4, EMVI and/or tumor deposits, threatened MRF or intersphincteric plane, and patient not eligible for sphincter-sparing surgery), the guidelines recommend considering neoadjuvant FOLFOX with selective addition of CRT in patients with insufficient response. The guidelines do not list alternative neoadjuvant options for this group of patients [65]. The ASCO guidelines acknowledge a different approach to MSI-H or dMMR rectal cancers and recommend upfront immunotherapy. Patients with dMMR rectal cancers who have contraindications to immunotherapy should be treated with the same approach as in pMMR patients [65].

The most recent update of the ASTRO guidelines concerning radiation therapy for rectal cancer was published in 2025 and the guidelines continue to frame recommendations around key questions [66]. The key questions 1 (indications for neoadjuvant RT) and 2 (selection of neoadjuvant regimens) related to neoadjuvant management options. The key recommendation for patients with stage II-III rectal cancer was to strongly consider neoadjuvant RT; however, among patients deemed at lower risk of locoregional recurrence, consideration of omission of neoadjuvant RT was conditionally recommended in patients agreeing to neoadjuvant chemotherapy with a favorable treatment response or who were interested in upfront surgery [66]. Lower risk was defined as cT2 or cT3a/b tumors > 5 cm from the anal verge, with <4 suspicious lymph nodes on baseline imaging (>1 cm in short axis), with mrCRM ≥ 2 mm and no mrEMVI, closely following PROSPECT clinical trial eligibility criteria. In patients who aim for non-operative management and organ preservations, radiation therapy should be a part of their neoadjuvant regimen [66]. The guidelines also included specific recommendations for people with dMMR/MSI-H rectal tumors, in whom the omission of neoadjuvant radiation therapy is recommended if they achieve cCR with upfront systemic therapy with checkpoint inhibitors [66].

ESMO also updated its practice guidelines for localized rectal cancer [67]. The guidelines include patients with cT1–cT3 localized rectal cancer in the upper third of the rectum as suitable candidates for avoiding neoadjuvant chemoradiation, with patients in the cT1N0/1 or cT2N0 group appropriate for upfront surgery and those with cT2N+ or cT3N0/N+ disease for either upfront surgery or neoadjuvant chemotherapy with CAPOX or FOLFOX with the option of adjuvant chemoradiation after the surgery if higher risk features are identified on pathology. In addition, the ESMO guidelines also include neoadjuvant chemotherapy or even upfront chemotherapy as management options in people with cT4 upper rectal tumors, even with N+ disease or mrMRF+ [67]. Similarly, patients with low and middle rectal tumors who are interested in proceeding with definitive surgery and who had cT1–3 N0–1 tumors were listed as appropriate for upfront surgery or neoadjuvant chemotherapy alone [67]. On the other hand, when organ preservation is intended, neoadjuvant radiation should be part of the neoadjuvant regimen [67].

Patients with dMMR or MSI-H rectal cancers should start with checkpoint inhibitor dostarlimab and in cases of cCR should proceed with the watch-and-wait approach. In patients with incomplete clinical response, salvage surgery should be offered [67].

The ESMO guidelines published in 2025 also focused on adjuvant management of patients who did not receive neoadjuvant radiation. They recommend offering adjuvant chemoradiation in cases of CRM positivity, pT4b tumors, pN2 disease, pN+ with extracapsular spread, proximity to the MRF and poor-quality TME [67]. Thus, most patients even with pT3 and/or pN1 disease do not require adjuvant radiation if these high-risk factors are absent [67].

At present, neoadjuvant radiation remains an important component of treatment for patients pursuing non-operative management and for those with indications for total neoadjuvant therapy (TNT).

## 8. Question(s) 7—What Are the Key Criteria to Guide Safe Implementation of a Non-Operative Watch-and-Wait Strategy in Rectal Cancer?

### Summary of Evidence

**Non-Operative Management of Rectal Cancer**

Evidence to support non-operative management of rectal cancer began two decades ago with the landmark paper by Habr-Gama published in *Annals of Surgery* in 2004, which followed a cohort of rectal cancer patients treated with long-course chemoradiation who achieved cCR post-therapy, and underwent either surgical or non-operative management, demonstrating similar disease-free and OS between groups [68].

Interest in NOM of rectal cancer led to the development of the International Watch & Wait Database (IWWD), an international multicenter registry that has provided information on real world outcomes of NOM rectal cancer strategies [69]. The initial publication, which reported longer term outcomes in *The Lancet* in 2018, described the outcomes of 1009 patients managed with a NOM protocol (87% with cCR) spanning 47 institutions across 15 countries, with a median follow-up of 3.3 years [69]. The 2-year cumulative incidence of regrowth in the cohort of complete responders was 25.2% (95% CI, 22.2–28.5) [69]. Most regrowths occurred within 2 years (88%) and of the local regrowths, 97% were within the bowel wall [69]. Distant metastases occurred in 8% of those with a cCR, and 5-year OS was 85% (95% CI, 80·9–87·7%) [69]. Although the strength of this dataset was real-world data describing the frequency and location of regrowths as well as survival outcomes, limitations included a lack of uniform neoadjuvant regimens and inclusion criteria, an unclear definition of complete responders (including timing of response), and non-uniform surveillance and surgical management approaches.

Then in 2022, the results of the Organ Preservation in Rectal Adenocarcinoma (OPRA) trial by Garcia-Aguilar et al. were published, which revolutionized rectal cancer treatment including the adoption of NOM [70]. This prospective multicenter RCT tested whether patients with stage II/III rectal cancer who were treated with TNT followed by either TME versus selective NOM (based on clinical response), would have better DFS when compared to a historical cohort that was treated with neoadjuvant chemoradiation (alone) followed by TME surgery and then adjuvant chemotherapy. The secondary outcome of the study was organ preservation (or TME-free survival). Patients were randomized to either undergo induction chemoradiation, followed by consolidation chemotherapy and reassessment (CRT-CNCT) or induction chemotherapy, followed by consolidation chemoradiation and then reassessment (INCT-CRT). The median follow-up was 3 years (interquartile range 1.84–4.06 years) and there was no difference in DFS between groups and the historical control. The rate of organ preservation at 3 years was 47% (95% CI, 41–53%) for the entire cohort. The 3-year probability of organ preservation was 77% (95% CI, 70–85%) for patients with a cCR and 40% (95% CI, 32–51%) for patients with a near-complete response (NCR) (*p*< 0.001) [71]. Although entering into a NOM protocol was common in both groups (66.5% INCT-CRT and 72.2% CRT-CNCT), regrowths were more common in the INCT-CRT (40%) compared to CRT-CNCT (27%) [70]. This initial data from the OPRA study supported the safety and feasibility of NOM after TNT. It also supported the preferential use of a CRT-CNCT protocol if organ preservation is desired.

A strength of the OPRA protocol was its use of a uniform definition for describing clinical responses as complete, near-complete, and incomplete using the Memorial Sloan Kettering Regression Schema, as well as a standardized timing of reassessment as 8 weeks (±4 weeks). Defining clinical response required digital rectal examination, endoscopy and MRI evaluation, and the schema from the Smith et al. paper is outlined below in Table 4 [72].

Longer-term results from the OPRA study were published in 2024, and median follow-up was 5.1 years (interquartile range, 3.5–5.7) [73]. There was no difference in 5-year DFS rates between groups, and these were 71% (95% CI, 64–79) for INCT-CRT and 69% for CRT-CNCT (95% CI, 62–77) (*p* = 0.68) [73]. TME-free survival was 39% (95% CI, 32–48) in the INCT-CRT group and 54% (95% CI, 46–62) in the CRT-CNCT group (*p* = 0.012) [73]. These long-term data further support the feasibility and safety of NOM and preference for CRT-CNCT if organ preservation is desired.

In the OPRA long-term cohort there were 81 (36%) patients with regrowth [73]. Of these regrowths, 94% occurred within 2 years and 99% occurred within 3 years [73]. DFS was similar for patients who underwent TME after restaging (64%, 95% CI, 53–78) and patients in NOM who underwent TME surgery after regrowth (64%, 95% CI, 53–78; *p* = 0.94) [73]. The combination of these findings supports the NOM strategy as safe and effective if appropriate surveillance is employed even if local regrowth occurs, as long it is followed by TME surgery. Rates and endoluminal location of local regrowth were similar to those reported in the IWWD2 [69]. The OPRA surveillance protocol is highlighted in the Table 5 below [70].

Also of importance is the risk of developing metastases after NOM. Further analysis of the IWWD, focusing on only those who developed a regrowth after NOM, identified the regrowth population at higher risk of developing distant metastases compared with those with no regrowth [74].

The OPRA consortium went on to publish several studies and secondary analyses to better understand predictors of success and safety of their NOM protocol. In the OPRA cohort, clinical tumor response grade was associated with improved organ preservation, DFS, LRFS, distant metastasis-free survival (MFS), and OS [71]. Patients with cCR had improved outcomes compared with those with NCR, which had improved outcomes compared with incomplete responses. Williams et al. evaluated baseline tumor characteristics to predict the need for TME surgery rather than NOM, and these were nodal disease (HR 1.66, 95% CI 1.12–2.48), extramural venous invasion (HR 1.57, 95% CI 1.07–2.29), mesorectal fascia involvement (HR 1.45, 95% CI 1.01–2.09), and tumor length (HR 1.11, 95% CI 1.00–1.22) [75]. Williams et al. also evaluated restaging MRIs and determined that the presence of restricted diffusion (OR 2.50; *p* = 0.013) and abnormal nodal morphologic features (OR 5.04; *p* = 0.02) at restaging were associated with residual disease [76]. Furthermore, Williams et al. evaluated patients with residual tumor on endoscopic assessment to determine endoscopic risk factors for residual tumor [77]. On multivariable regression analysis, several characteristics of an NCR including ulceration (OR 6.66, 95% CI 2.54–19.9), irregular mucosa (OR 3.66, 95% CI 1.61–8.68), and nodularity (OR 2.96, 95% CI 1.36–6.58) were predictors of residual tumor, whereas a flat scar was associated with lower odds of having residual disease (OR 0.32, 95% CI 0.11–0.93) [77]. In summary, both MRI and endoscopy are valuable tools to identify those most likely to enter a NOM and achieve a cCR. Those with more aggressive baseline tumors on MRI are less likely to enter NOM. Those with cCR after total neoadjuvant treatment have better oncologic outcomes compared to those with NCR and incomplete clinical response.

To determine the effectiveness of surveillance modalities on identifying regrowth, a single-center study evaluated those with suspected local tumor regrowth during surveillance with either MRI or endoscopy [78]. There were 112 patients with suspected regrowth of which 99 underwent surgery [78]. For those with abnormal MRI but normal endoscopy who underwent surgery, pathological complete response was 44% (4/9) [78]. For those with abnormal endoscopy but normal MRI, pCR was 18% (6/34), and for those with abnormal endoscopy and MRI, pCR was 4% (2/56) [78]. Their conclusion was that normal endoscopy during surveillance was significantly associated with higher odds of pathological complete response (OR 8.2, *p*  =  0.012) whereas normal MRI showed a non-significant association (OR 2.11, *p*  =  0.33) [78]. These findings suggest that when isolated abnormal MRI findings are identified during surveillance, repeat radiologic evaluation prior to proceeding to surgery may help minimize unnecessary interventions [78].

Another single-center study evaluated a retrospective cohort, “natural experiment” during COVID when short-course radiation was used more often in total neoadjuvant therapy protocols [79]. Similar rates of cCR were observed between those who received short-course radiation versus long-course chemoradiation. However, in patients managed with NOM, long-course chemoradiation resulted in higher 2-year organ preservation (89%, 95% CI 83–95% vs. 70%, 95% CI 55–90%; *p* = 0.005) and lower 2-year local regrowth (19%, 95% CI 11–26% vs. 36%, 95% CI 16% to 52%; *p* = 0.072) compared with short-course radiation [79]. These data suggest that long-course chemoradiation may be preferred over short-course radiation, when organ preservation is desired following TNT.

The IWWD data was analyzed further to determine differences in outcomes for those who had an initial cCR versus those with NCR who subsequently developed cCR on later assessment. The 2-year organ preservation rate was similar between groups: 77.8% (95% CI 74.2–81.5) and 79.3% (95% CI 75.1–83.7), respectively (*p* = 0.499). Similarly, no differences were identified for distant MFS or OS [80]. These findings support the inclusion of both NCR and cCR within NOM protocols similar to the OPRA study protocol. Nevertheless, patients with NCR managed with an initial NOM approach should undergo short-interval restaging, and TME surgery should be considered in cases of progression or failure to achieve cCR [81].

## Figures and Tables

**Table 1:** Attendees at the 27th Annual WCGCCC.

Name	Specialty	Organization
Aswin Abraham	Radiation Oncologist	Alberta Health Services
Sean Addison	Medical Oncologist	BC Cancer
Shahid Ahmed	Medical Oncologist	Saskatchewan Cancer Agency
Osama Ahmed	Medical Oncologist	Saskatoon Cancer Centre
Adrian Box	Molecular Pathology	Alberta Precision Laboratories
Haji Chalchal	Medical Oncologist	Allan Blair Cancer Ctr
Theresa Chan	Medical Oncologist	BC Cancer
Navdeep Dehar	Medical Oncologist	Arthur J.E. Child Comprehensive Cancer Center
Jay Easaw	Medical Oncologist	Cross Cancer Institute
Bharath Gangadharaiah	Medical Oncologist	CancerCare Manitoba
Georgia Geller	Medical Oncologist	BC Cancer
AJ Ghose	Radiation Oncologist	Alberta Health Services
Jiti Gill	Medical Oncologist	BC Cancer
Sharlene Gill	Medical Oncologist	BC Cancer
Tamara Gimon	Colorectal Surgeon	Alberta Health Services
Vallerie Gordon	Medical Oncologist	Saskatchewan Cancer Agency
Branawan Gowrishankar	Medical Oncologist	Cross Cancer Institute
Petra Grendarova	Radiation Oncologist	BC Cancer
Safiya Karim	Medical Oncologist	Arthur J.E. Child Comprehensive Cancer Center
Christina Kim	Medical Oncologist	CancerCare Manitoba
Mark Kristjanson	Family Physician in Oncology	CancerCare Manitoba
Hannah Latour	Resident	University of Alberta
Duc Le	Radiation Oncologist	Saskatoon Cancer Centre
Richard Lee-Ying	Medical Oncologist	Arthur J.E. Child Comprehensive Cancer Center
Shaun Loewen	Radiation Oncologist	Arthur J.E. Child Comprehensive Cancer Center
Karen Mulder	Medical Oncologist	Cross Cancer Institute
James Paul	Medical Oncologist	University of Manitoba & CancerCare Manitoba
Renata Peixoto	Medical Oncologist	BC Cancer
Scott Strum	Medical Oncologist	Arthur J.E. Child Comprehensive Cancer Center
Hui Wang	Pathologist	Saskatchewan Health Authority
Ralph Wong	Medical Oncologist	CancerCare Manitoba
Simon Yu	Medical Oncologist	Burnaby Hospital Cancer Centre

**Table 2:** Clinical questions addressing specific aspects of interest in colorectal cancer are addressed as part of the 27th annual WCGCCC.

1. Exercise as an Adjuvant Strategy in Early-stage Colon Cancer: The CHALLENGE Trial Which patients with resected colon cancer should be recommended structured exercise programs to improve outcomes? What key components of a structured exercise program should be offered to patients with resected colorectal cancer?
2. Precision Adjuvant Therapy for Phosphatidylinositol 3-Kinase (PI3K) Pathway Mutated Colorectal Cancer: The ALASCCA Trial 2a—What is the optimal testing strategy for screening for PI3K alterations? What alternative testing strategies can be considered? 2b—Which patients with resected colorectal cancer should be offered adjuvant ASA? In the absence of PI3K testing, can adjuvant ASA be offered to all cases of resected colorectal cancer?
3. Adjuvant Immune Checkpoint Inhibition in dMMR Stage III Colon Cancer: The ATOMIC Trial Is there a role for immune checkpoint inhibitors in addition to adjuvant chemotherapy for patients with resected stage III dMMR colon cancer? Is there an optimal time for immune checkpoint inhibition pre-, post- or perioperatively?
4. BREAKWATER: BRAF-Directed Therapy in BRAF V600E-mutatated Metastatic Colorectal Cancer What is the optimal first-line therapy for patients with BRAF V600E-mutant metastatic colorectal cancer?
5. Optimizing Immunotherapy in dMMR Metastatic Colorectal Cancer: CheckMate-8HW What is the preferred immunotherapy approach for patients with dMMR metastatic colorectal cancer: single-agent or combination immunotherapy?
6. Radiotherapy in Rectal Cancer: Can It Be Safely Omitted in Selected Patients? In which patients with localized rectal cancer can radiotherapy be safely omitted without compromising outcomes?
7. Non-Operative Management of Rectal Cancer What are the key criteria to guide safe implementation of a non-operative watch-and-wait strategy in rectal cancer?

**Table 4:** Memorial Sloan Kettering Regression Schema [72] ^a^.

	Complete Response	Near-Complete Response	Incomplete Response
Endoscopy	Flat, white scar Telangiectasia No ulcer No nodularity	Irregular mucosa Small mucosal nodules or minor mucosal abnormality Superficial ulceration Mild persisting erythema of the scar	Visible tumor
Digital Rectal Exam	Normal	Smooth induration or minor mucosal abnormalities	Palpable tumor nodules
MRI-T2W	Only dark T2 signal, no intermediate T2 signal AND No visible lymph nodes	Mostly dark T2 signal, some remaining intermediate signal AND/OR Partial regression of lymph nodes	More intermediate than dark T2 signal, no T2 scar AND/OR No regression of lymph nodes
MRI-DW	No visible tumor on B800-B1000 signal AND/OR Lack of or low signal on ADC map Uniform, linear signal in wall above tumor is ok	Significant regression of signal on B800-B1000 AND/OR Minimal or low residual signal on ADC map	Insignificant regression of signal on B800-B1000 AND/OR Obvious low signal on ADC

^a^ ADC, apparent diffusion coefficient.

**Table 5:** OPRA surveillance protocol [70] ^b^.

Years	Year 1	Year 2	Year 3	Year 4	Year 5
DRE, flex sig, CEA	Q 4 months	Q 4 months	Q 6 months	Q 6 months	Q 6 months
MRI	Q 6 months	Q 6 months	Q 12 months	Q 12 months	Q 12 months
CT CAP	once	once	once	once	once
Colonoscopy	once	--	--	--	once

^b^ DRE, digital rectal exam; flex sig, flexible sigmoidoscopy; CEA, carcinoembryonic antigen; MRI, magnetic resonance imaging; CT CAP, computed tomography of chest abdomen and pelvis.

## Data Availability

The data presented in this study are available in this article.

## References

[1] Markozannes G., Becerra-Tomás N., Cariolou M., Balducci K., Vieira R., Kiss S., Aune D., Greenwood D.C., Gunter M.J., Copson E. (2024). Post-diagnosis physical activity and sedentary behaviour and colorectal cancer prognosis: A Global Cancer Update Programme (CUP Global) systematic literature review and meta-analysis. Int. J. Cancer.

[2] Brown J.C., Ma C., Shi Q., Fuchs C.S., Meyer J., Niedzwiecki D., Zemla T., Couture F., Kuebler P., Kumar P. (2023). Physical Activity in Stage III Colon Cancer: CALGB/SWOG 80702 (Alliance). J. Clin. Oncol..

[3] Courneya K.S., Vardy J.L., O’Callaghan C.J., Gill S., Friedenreich C.M., Wong R.K.S., Dhillon H.M., Coyle V., Chua N.S., Jonker D.J. (2025). Structured Exercise after Adjuvant Chemotherapy for Colon Cancer. N. Engl. J. Med..

[4] Ajzen I. (1991). The theory of planned behavior. Organ. Behav. Hum. Decis. Process..

[5] Martling A., Hed Myrberg I., Nilbert M., Grönberg H., Granath F., Eklund M., Öresland T., Iversen L.H., Haapamäki C., Janson M. (2025). Low-Dose Aspirin for PI3K-Altered Localized Colorectal Cancer. N. Engl. J. Med..

[6] Baron J.A., Cole B.F., Sandler R.S., Haile R.W., Ahnen D., Bresalier R., McKeown-Eyssen G., Summers R.W., Rothstein R., Burke C.A. (2003). A Randomized Trial of Aspirin to Prevent Colorectal Adenomas. N. Engl. J. Med..

[7] Din F.V.N., Theodoratou E., Farrington S.M., Tenesa A., Barnetson R.A., Cetnarskyj R., Stark L., Porteous M.E., Campbell H., Dunlop M.G. (2010). Effect of aspirin and NSAIDs on risk and survival from colorectal cancer. Gut.

[8] Flossmann E., Rothwell P.M. (2007). Effect of aspirin on long-term risk of colorectal cancer: Consistent evidence from randomised and observational studies. Lancet.

[9] Algra A.M., Rothwell P.M. (2012). Effects of regular aspirin on long-term cancer incidence and metastasis: A systematic comparison of evidence from observational studies versus randomised trials. Lancet Oncol..

[10] Burn J., Gerdes A.M., MacRae F., Mecklin J.P., Moeslein G., Olschwang S., Eccles D., Evans D.G., Maher E.R., Bertario L. (2011). Long-term effect of aspirin on cancer risk in carriers of hereditary colorectal cancer: An analysis from the CAPP2 randomised controlled trial. Lancet.

[11] Dubé C., Rostom A., Lewin G., Tsertsvadze A., Barrowman N., Code C., Sampson M., Moher D. (2007). The use of aspirin for primary prevention of colorectal cancer: A systematic review prepared for the U.S. Preventive Services Task Force. Ann. Intern. Med..

[12] Chan A.T., Ogino S., Fuchs C.S. (2009). Aspirin use and survival after diagnosis of colorectal cancer. JAMA.

[13] Rothwell P.M., Wilson M., Elwin C.E., Norrving B., Algra A., Warlow C.P., Meade T.W. (2010). Long-term effect of aspirin on colorectal cancer incidence and mortality: 20-year follow-up of five randomised trials. Lancet.

[14] Uddin S., Ahmed M., Hussain A., Assad L., Al-Dayel F., Bavi P., Al-Kuraya K.S., Munkarah A. (2010). Cyclooxygenase-2 inhibition inhibits PI3K/AKT kinase activity in epithelial ovarian cancer. Int. J. Cancer..

[15] Kaur J., Sanyal S.N. (2010). PI3-kinase/Wnt association mediates COX-2/PGE2 pathway to inhibit apoptosis in early stages of colon carcinogenesis: Chemoprevention by diclofenac. Tumour Biol..

[16] Liao X., Lochhead P., Nishihara R., Morikawa T., Kuchiba A., Yamauchi M., Imamura Y., Qian Z.R., Baba Y., Shima K. (2012). Aspirin Use, Tumor PIK3CA Mutation, and Colorectal-Cancer Survival. N. Engl. J. Med..

[17] Chia J.W.K., Segelov E., Deng Y., Ho G.F., Wang W., Han S., Sharma A., Ding K., Chen G., Jeffery M.G. (2025). Aspirin after completion of standard adjuvant therapy for colorectal cancer (ASCOLT): An international, multicentre, phase 3, randomised, double-blind, placebo-controlled trial. Lancet Gastroenterol. Hepatol..

[18] Güller U., Hayoz S., Horber D., Jochum W., De Dosso S., Koeberle D., Schacher S., Inauen R., Stahl M., Delaunoit T. (2025). Adjuvant Aspirin Treatment in PIK3CA-Mutated Colon Cancer Patients: The SAKK 41/13 Prospective Randomized Placebo-Controlled Double-Blind Trial. Clin. Cancer. Res..

[19] DeWitt J.T., Jimenez-Tovar D., Mazumder A., Haricharan S. (2025). Advances in diagnostic and therapeutic applications of mismatch repair loss in cancer. DNA Repair..

[20] Sun M., Monahan K., Moquet J., Barnard S. (2025). Colorectal cancers associated with mismatch repair deficiency. Front. Med..

[21] Tran B., Kopetz S., Tie J., Gibbs P., Jiang Z.Q., Lieu C.H., Agarwal A., Maru D.M., Sieber O., Desai J. (2011). Impact of BRAF mutation and microsatellite instability on the pattern of metastatic spread and prognosis in metastatic colorectal cancer. Cancer.

[22] Jin Z., Sinicrope F.A. (2022). Mismatch Repair-Deficient Colorectal Cancer: Building on Checkpoint Blockade. J. Clin. Oncol..

[23] Ribic C.M., Sargent D.J., Moore M.J., Thibodeau S.N., French A.J., Goldberg R.M., Hamilton S.R., Laurent-Puig P., Gryfe R., Shepherd L.E. (2003). Tumor Microsatellite-Instability Status as a Predictor of Benefit from Fluorouracil-Based Adjuvant Chemotherapy for Colon Cancer. N. Engl. J. Med..

[24] Sargent D.J., Marsoni S., Monges G., Thibodeau S.N., Labianca R., Hamilton S.R., French A.J., Kabat B., Foster N.R., Torri V. (2010). Defective mismatch repair as a predictive marker for lack of efficacy of fluorouracil-based adjuvant therapy in colon cancer. J. Clin. Oncol..

[25] Cohen R., Taieb J., Fiskum J., Yothers G., Goldberg R., Yoshino T., Alberts S., Allegra C., De Gramont A., Seitz J.F. (2021). Microsatellite Instability in Patients with Stage III Colon Cancer Receiving Fluoropyrimidine with or Without Oxaliplatin: An ACCENT Pooled Analysis of 12 Adjuvant Trials. J. Clin. Oncol..

[26] Tomasello G., Ghidini M., Galassi B., Grossi F., Luciani A., Petrelli F. (2022). Survival benefit with adjuvant chemotherapy in stage III microsatellite-high/deficient mismatch repair colon cancer: A systematic review and meta-analysis. Sci. Rep..

[27] National Comprehensive Cancer Network [Internet] (2025). NCCN Clinical Practice Guidelines in Oncology (NCCN Guidelines^®^): Colon Cancer (Version 5.2025). https://www.nccn.org/guidelines/category_1.

[28] Argilés G., Tabernero J., Labianca R., Hochhauser D., Salazar R., Iveson T., Laurent-Puig P., Quirke P., Yoshino T., Taieb J. (2020). Localised colon cancer: ESMO Clinical Practice Guidelines for diagnosis, treatment and follow-up^†^. Ann. Oncol..

[29] Le D.T., Durham J.N., Smith K.N., Wang H., Bartlett B.R., Aulakh L.K., Lu S., Kemberling H., Wilt C., Luber B.S. (2017). Mismatch repair deficiency predicts response of solid tumors to PD-1 blockade. Science.

[30] André T., Shiu K.K., Kim T.W., Jensen B.V., Jensen L.H., Punt C.J.A., Smith D., Garcia-Carbonero R., Alcaide-Garcia J., Gibbs P. (2025). Pembrolizumab versus chemotherapy in microsatellite instability-high or mismatch repair-deficient metastatic colorectal cancer: 5-year follow-up from the randomized phase III KEYNOTE-177 study. Ann. Oncol..

[31] André T., Elez E., Van Cutsem E., Jensen L.H., Bennouna J., Mendez G., Schenker M., de la Fouchardiere C., Limon M.L., Yoshino T. (2024). Nivolumab plus Ipilimumab in Microsatellite-Instability–High Metastatic Colorectal Cancer. N. Engl. J. Med..

[32] Sinicrope F.A., Ou F.-S., Arnold D., Peters W.R., Behrens R.J., Lieu C.H., Matin K., Cohen D.J., Potter S.L., Nixon A.B. (2026). Atezolizumab plus FOLFOX for Stage III Mismatch Repair-Deficient Colon Cancer. N. Engl. J. Med..

[33] Sinicrope F.A., Turk M.J. (2024). Immune checkpoint blockade: Timing is everything. J. Immunother. Cancer.

[34] Topalian S.L., Taube J.M., Pardoll D.M. (2020). Neoadjuvant checkpoint blockade for cancer immunotherapy. Science.

[35] Chalabi M., Fanchi L.F., Dijkstra K.K., Van den Berg J.G., Aalbers A.G., Sikorska K., Lopez-Yurda M., Grootscholten C., Beets G.L., Snaebjornsson P. (2020). Neoadjuvant immunotherapy leads to pathological responses in MMR-proficient and MMR-deficient early-stage colon cancers. Nat. Med..

[36] Chalabi M., Verschoor Y.L., Tan P.B., Balduzzi S., Van Lent A.U., Grootscholten C., Dokter S., Büller N.V., Grotenhuis B.A., Kuhlmann K. (2024). Neoadjuvant Immunotherapy in Locally Advanced Mismatch Repair–Deficient Colon Cancer. N. Engl. J. Med..

[37] Shiu K.K., Jiang Y., Saunders M., Seligmann J.F., Iveson T., Wilson R.H., Graham J.S., Khan K.H., Militello A.M., Irvine S. (2024). NEOPRISM-CRC: Neoadjuvant pembrolizumab stratified to tumour mutation burden for high-risk stage 2 or stage 3 deficient-MMR/MSI-high colorectal cancer. Proceedings of the 2024 ASCO Annual Meeting II, Chicago, IL, USA, 31 May–4 June 2024.

[38] Qvortrup C., Justesen T.F., Tarpgaard L.S., Bulut M., Hansen T., Rahr H.B., Petersen P.C., Eriksen J.R., Salomon S., Gotschalck K.A. (2025). Single-cycle neoadjuvant pembrolizumab in patients with stage I-III MMR-deficient colon cancer: Final analysis of the RESET-C study. Proceedings of the 2025 ASCO Gastrointestinal Cancers Symposium, San Francisco, CA, USA, 23–25 January 2025.

[39] Cercek A., Lumish M., Sinopoli J., Weiss J., Shia J., Lamendola-Essel M., El Dika I.H., Segal N., Shcherba M., Sugarman R. (2022). PD-1 Blockade in Mismatch Repair–Deficient, Locally Advanced Rectal Cancer. N. Engl. J. Med..

[40] Tie J., Gibbs P., Lipton L., Christie M., Jorissen R.N., Burgess A.W., Croxford M., Jones I., Langland R., Kosmider S. (2011). Optimizing targeted therapeutic development: Analysis of a colorectal cancer patient population with the BRAFV600E mutation. Int. J. Cancer.

[41] Elez E., Yoshino T., Shen L., Lonardi S., Van Cutsem E., Eng C., Kim T.W., Wasan H.S., Desai J., Ciardiello F. (2025). Encorafenib, Cetuximab, and mFOLFOX6 in BRAF -Mutated Colorectal Cancer. N. Engl. J. Med..

[42] Kopetz S., Yoshino T., Van Cutsem E., Eng C., Kim T.W., Wasan H.S., Desai J., Ciardiello F., Yaeger R., Maughan T.S. BREAKWATER: Analysis of first-line encorafenib + cetuximab + chemotherapy in BRAF V600E-mutant metastatic colorectal cancer. Proceedings of the 2025 ASCO Gastrointestinal Cancers Symposium.

[43] Kopetz S., Wasan H.S., Yoshino T., Kim T.W., Eng C., Van Cutsem E., Ciardiello F., Desai J., Maughan T.S., Yaeger R. (2026). BREAKWATER: Primary analysis of first-line (1L) encorafenib + cetuximab (EC) + FOLFIRI in BRAF V600E-mutant metastatic colorectal cancer (mCRC). J. Clin. Oncol..

[44] Sinicrope F.A., Sargent D.J. (2012). Molecular pathways: Microsatellite instability in colorectal cancer: Prognostic, predictive, and therapeutic implications. Clin. Cancer Res..

[45] André T., Shiu K.K., Kim T.W., Jensen B.V., Jensen L.H., Punt C., Smith D., Garcia-Carbonero R., Benavides M., Gibbs P. (2020). Pembrolizumab in Microsatellite-Instability–High Advanced Colorectal Cancer. N. Engl. J. Med..

[46] Andre T., Amonkar M., Norquist J.M., Shiu K.K., Kim T.W., Jensen B.V., Jensen L.H., Punt C.J.A., Smith D., Garcia-Carbonero R. (2021). Health-related quality of life in patients with microsatellite instability-high or mismatch repair deficient metastatic colorectal cancer treated with first-line pembrolizumab versus chemotherapy (KEYNOTE-177): An open-label, randomised, phase 3 trial. Lancet Oncol..

[47] André T., Elez E., Lenz H.J., Jensen L.H., Touchefeu Y., Van Cutsem E., Garcia-Carbonero R., Tougeron D., Mendez G.A., Schenker M. (2025). Nivolumab plus ipilimumab versus nivolumab in microsatellite instability-high metastatic colorectal cancer (CheckMate 8HW): A randomised, open-label, phase 3 trial. Lancet.

[48] Folkesson J., Birgisson H., Pahlman L., Cedermark B., Glimelius B., Gunnarsson U. (2005). Swedish rectal cancer trial: Long lasting benefits from radiotherapy on survival and local recurrence rate. J. Clin. Oncol..

[49] Roh M.S., Colangelo L.H., O’Connell M.J., Yothers G., Deutsch M., Allegra C.J., Kahlenberg M.S., Baez-Diaz L., Ursiny C.S., Petrelli N.J. (2009). Preoperative multimodality therapy improves disease-free survival in patients with carcinoma of the rectum: NSABP R-03. J. Clin. Oncol..

[50] Sauer R., Liersch T., Merkel S., Fietkau R., Hohenberger W., Hess C., Becker H., Raab H.R., Villanueva M.T., Witzigmann H. (2012). Preoperative versus postoperative chemoradiotherapy for locally advanced rectal cancer: Results of the German CAO/ARO/AIO-94 randomized phase III trial after a median follow-up of 11 years. J. Clin. Oncol..

[51] Sebag-Montefiore D., Stephens R.J., Steele R., Monson J., Grieve R., Khanna S., Quirke P., Couture J., de Metz C., Myint A.S. (2009). Preoperative radiotherapy versus selective postoperative chemoradiotherapy in patients with rectal cancer (MRC CR07 and NCIC-CTG C016): A multicentre, randomised trial. Lancet.

[52] Van Gijn W., Marijnen C.A.M., Nagtegaal I.D., Kranenbarg E.M.K., Putter H., Wiggers T., Rutten H.J.T., Påhlman L., Glimelius B., Van de Velde C.J.H. (2011). Preoperative radiotherapy combined with total mesorectal excision for resectable rectal cancer: 12-year follow-up of the multicentre, randomised controlled TME trial. Lancet Oncol..

[53] Abraha I., Aristei C., Palumbo I., Lupattelli M., Trastulli S., Cirocchi R., De Florio R., Valentini V. (2018). Preoperative radiotherapy and curative surgery for the management of localised rectal carcinoma. Cochrane Database Syst. Rev..

[54] Camma C., Giunta M., Fiorica F., Pagliaro L., Craxi A., Cottone M. (2000). Preoperative radiotherapy for resectable rectal cancer: A meta-analysis. JAMA.

[55] Rahbari N.N., Elbers H., Askoxylakis V., Motschall E., Bork U., Bü Chler M.W., Weitz J., Koch M. (2013). Neoadjuvant radiotherapy for rectal cancer: Meta-analysis of randomized controlled trials. Ann. Surg. Oncol..

[56] Peeters K.C.M.J., Marijnen C.A.M., Nagtegaal I.D., Kranenbarg E.K., Putter H., Wiggers T., Rutten H., Pahlman L., Glimelius B., Leer J.W. (2007). The TME trial after a median follow-up of 6 years: Increased local control but no survival benefit in irradiated patients with resectable rectal carcinoma. Ann. Surg..

[57] Glynne-Jones R., Wyrwicz L., Tiret E., Brown G., Rödel C., Cervantes A., Arnold D. (2017). Rectal cancer: ESMO Clinical Practice Guidelines for diagnosis, treatment and follow-up. Ann. Oncol..

[58] Wo J.Y., Anker C.J., Ashman J.B., Bhadkamkar N.A., Bradfield L., Chang D.T., Dorth J., Garcia-Aguilar J., Goff D., Jacqmin D. (2021). Radiation Therapy for Rectal Cancer: Executive Summary of an ASTRO Clinical Practice Guideline. Pract. Radiat. Oncol..

[59] Schrag D., Shi Q., Weiser M.R., Gollub M.J., Saltz L.B., Musher B.L., Goldberg J., Baghdadi T.A., Goodman K.A., McWilliams R.R. (2023). Preoperative Treatment of Locally Advanced Rectal Cancer. N. Engl. J. Med..

[60] Basch E., Dueck A.C., Mitchell S.A., Mamon H., Weiser M., Saltz L., Gollub M., Rogak L., Ginos B., Mazza G.L. (2023). Patient-Reported Outcomes during and after Treatment for Locally Advanced Rectal Cancer in the PROSPECT Trial (Alliance N1048). J. Clin. Oncol..

[61] Mei W.J., Wang X.Z., Li Y.F., Sun Y.M., Yang C.K., Lin J.Z., Wu Z.G., Zhang R., Wang W., Li Y. (2023). Neoadjuvant Chemotherapy with CAPOX Versus Chemoradiation for Locally Advanced Rectal Cancer with Uninvolved Mesorectal Fascia (CONVERT): Initial Results of a Phase III Trial. Ann. Surg..

[62] Ding P.R., Wang X.Z., Li Y.-F., Sun Y.M., Yang C.K., Wu Z.G., Zhang R., Wang W., Li Y., Zhuang Y.Z. (2023). LBA26 Neoadjuvant chemotherapy with CAPOX versus chemoradiation for locally advanced rectal cancer with uninvolved mesorectal fascia (CONVERT): Final results of a phase III trial. Ann. Oncol..

[63] Deng Y., Chi P., Lan P., Wang L., Chen W., Cui L., Chen D., Cao J., Wei H., Peng X. (2019). Neoadjuvant modified folfox6 with or without radiation versus fluorouracil plus radiation for locally advanced rectal cancer: Final results of the Chinese FOWARC trial. J. Clin. Oncol..

[64] Zhang J., Chi P., Shi L., Cui L., Gao J., Li W., Wei H., Cheng L., Huang Z., Cai G. (2024). Neoadjuvant Modified Infusional Fluorouracil, Leucovorin, and Oxaliplatin with or Without Radiation Versus Fluorouracil Plus Radiation for Locally Advanced Rectal Cancer: Updated Results of the FOWARC Study After a Median Follow-Up of 10 Years. J. Clin. Oncol..

[65] Scott A.J., Kennedy E.B., Berlin J., Brown G., Chalabi M., Cho M.T., Cusnir M., Dorth J., George M., Kachnic L.A. (2024). Management of Locally Advanced Rectal Cancer: ASCO Guideline. J. Clin. Oncol..

[66] Wo J.Y., Ashman J.B., Bhadkamkar N.A., Bradfield L., Chang D.T., Hanna N., Hawkins M., Holtz M., Kim E., Kelly P. (2025). Radiation Therapy for Rectal Cancer: An ASTRO Clinical Practice Guideline Focused Update. Pract. Radiat. Oncol..

[67] Hofheinz R.D., Fokas E., Benhaim L., Price T.J., Arnold D., Beets-Tan R., Guren M.G., Hospers G.A.P., Lonardi S., Nagtegaal I.D. (2025). Localised rectal cancer: ESMO Clinical Practice Guideline for diagnosis, treatment and follow-up. Ann. Oncol..

[68] Habr-Gama A., Perez R.O., Nadalin W., Sabbaga J., Ribeiro U., Silva E Sousa A.H., Campos F.G., Kiss D.R., Gama-Rodrigues J., Michelassi F. (2004). Operative versus nonoperative treatment for stage 0 distal rectal cancer following chemoradiation therapy: Long-term results. Ann. Surg..

[69] van der Valk M.J.M., Hilling D.E., Bastiaannet E., Meershoek-Klein Kranenbarg E., Beets G.L., Figueiredo N.L., Habr-Gama A., Perez R.O., Renehan A.G., van de Velde C.J.H. (2018). Long-term outcomes of clinical complete responders after neoadjuvant treatment for rectal cancer in the International Watch & Wait Database (IWWD): An international multicentre registry study. Lancet.

[70] Garcia-Aguilar J., Patil S., Gollub M.J., Kim J.K., Yuval J.B., Thompson H.M., Verheij F.S., Omer D.M., Lee M., Dunne R.F. (2022). Organ Preservation in Patients with Rectal Adenocarcinoma Treated with Total Neoadjuvant Therapy. J. Clin. Oncol..

[71] Thompson H.M., Omer D.M., Lin S., Kim J.K., Yuval J.B., Verheij F.S., Qin L.X., Gollub M.J., Wu A.J.C., Lee M. (2024). Organ Preservation and Survival by Clinical Response Grade in Patients with Rectal Cancer Treated with Total Neoadjuvant Therapy A Secondary Analysis of the OPRA Randomized Clinical Trial. JAMA Netw. Open.

[72] Smith J.J., Chow O.S., Gollub M.J., Nash G.M., Temple L.K., Weiser M.R., Guillem J.G., Paty P.B., Avila K., Garcia-Aguilar J. (2015). Organ Preservation in Rectal Adenocarcinoma: A phase II randomized controlled trial evaluating 3-year disease-free survival in patients with locally advanced rectal cancer treated with chemoradiation plus induction or consolidation chemotherapy, and total mesorectal excision or nonoperative management. BMC Cancer.

[73] Verheij F.S., Omer D.M., Williams H., Lin S.T., Qin L.X., Buckley J.T., Thompson H.M., Yuval J.B., Kim J.K., Dunne R.F. (2024). Long-Term Results of Organ Preservation in Patients with Rectal Adenocarcinoma Treated with Total Neoadjuvant Therapy: The Randomized Phase II OPRA Trial. J. Clin. Oncol..

[74] Fernandez L.M., São Julião G.P., Renehan A.G., Beets G.L., Papoila A.L., Vailati B.B., Bahadoer R.R., Kranenbarg E.M.K., Roodvoets A.G.H., Figueiredo N.L. (2023). The Risk of Distant Metastases in Patients with Clinical Complete Response Managed by Watch and Wait after Neoadjuvant Therapy for Rectal Cancer: The Influence of Local Regrowth in the International Watch and Wait Database. Dis. Colon Rectum.

[75] Williams H., Yuval J.B., Verheij F.S., Miranda J., Lin S.T., Omer D.M., Qin L.X., Gollub M.J., Kim T.H., Garcia-Aguilar J. (2024). Baseline MRI predictors of successful organ preservation in the Organ Preservation in Rectal Adenocarcinoma (OPRA) trial. Br. J. Surg..

[76] Williams H., Omer D.M., Thomson H.M., Lin S.T., Verheij F.S., Miranda J., Yuval J.B., Buckley J., Marco M.R., Qin L.X. (2024). MRI Predicts Residual Disease and Outcomes in Watch-and-Wait Patients with Rectal Cancer. Radiology.

[77] Williams H., Thompson H.M., Lin S.T., Verheij F.S., Omer D.M., Qin L.X., Garcia-Aguilar J. (2024). Endoscopic Predictors of Residual Tumor after Total Neoadjuvant Therapy: A Post Hoc Analysis from the Organ Preservation in Rectal Adenocarcinoma Trial. Dis. Colon Rectum.

[78] Yeung T.M., Rosen R.Y., Bercz A., Williams H., Omer D., Verheij F.S., Behman R., Marcadis A., Shia J., Cercek A. (2025). The value of MRI and flexible sigmoidoscopy in detecting tumor regrowth in watch and wait rectal cancer patients undergoing active surveillance after total neoadjuvant therapy. Surg. Endosc..

[79] Bercz A., Park B.K., Pappou E., Nemirovsky D., Sarkar R., Yamner M., Omer D., Verheij F.S., Alvarez J., Atri P. (2024). Organ preservation after neoadjuvant long-course chemoradiotherapy versus short-course radiotherapy. Ann. Oncol..

[80] Temmink S.J.D., Peeters K.C.M.J., Bahadoer R.R., Kranenbarg E.M.K., Roodvoets A.G.H., Melenhorst J., Burger J.W.A., Wolthuis A., Renehan A.G., Figueiredo N.L. (2023). Watch and wait after neoadjuvant treatment in rectal cancer: Comparison of outcomes in patients with and without a complete response at first reassessment in the International Watch & Wait Database (IWWD). Br. J. Surg..

[81] Williams H., Lee C., Garcia-Aguilar J. (2024). Nonoperative management of rectal cancer. Front. Oncol..

